# AMS-MLP: adaptive multi-scale MLP network with multi-scale context relation decoder for pepper leaf segmentation

**DOI:** 10.3389/fpls.2025.1515105

**Published:** 2025-04-08

**Authors:** Jiangxiong Fang, Huaxiang Liu, Shiqing Zhang, Hui Hu, Huaqi Gu, Youyao Fu

**Affiliations:** ^1^ Institute of Intelligent Information Processing, Taizhou University, Taizhou, Zhejiang, China; ^2^ Department of Information and Remote Sensing, Jiangxi Provincial Natural Resources Development Center, Nanchang, Jiangxi, China

**Keywords:** pepper leaf segmentation, multi-scale MLP, multi-path aggregation module, context relation mask module, adaptive attention mechanism

## Abstract

**Introduction:**

Pepper leaf segmentation plays a pivotal role in monitoring pepper leaf diseases across diverse backgrounds and ensuring healthy pepper growth. However, existing Transformer-based segmentation methods grapple with computational inefficiency, excessive parameterization, and inadequate utilization of edge information.

**Methods:**

To address these challenges, this study introduces an Adaptive Multi-Scale MLP (AMS-MLP) framework. This framework integrates the Multi-Path Aggregation Module (MPAM) and the Multi-Scale Context Relation Mask Module (MCRD) to refine object boundaries in pepper leaf segmentation. The AMS-MLP includes an encoder, an Adaptive Multi-Scale MLP (AM-MLP) module, and a decoder. The encoder’s MPAM fuses five-scale features for accurate boundary extraction. The AM-MLP splits features into global and local branches, with an adaptive attention mechanism balancing them. The decoder enhances boundary feature extraction using MCRD.

**Results:**

To validate the proposed method, extensive experiments were conducted on three pepper leaf datasets with varying backgrounds. Results demonstrate mean Intersection over Union (mIoU) scores of 97.39%, 96.91%, and 97.91%, and F1 scores of 98.29%, 97.86%, and 98.51% across the datasets, respectively.

**Discussion:**

Comparative analysis with U-Net and state-of-the-art models reveals that the proposed method significantly improves the accuracy and efficiency of pepper leaf image segmentation.

## 1 Introduction

Pepper is a crucial crop in global agriculture, with China being the largest producer and consumer, accounting for 37% of the world’s pepper planting area. Essential for daily consumption, pepper plants are highly susceptible to diseases, particularly those affecting the leaves, leading to significant economic losses if not promptly detected and controlled (Bhavini and Sheshang, 2015; Cruz et al., 2019). In practice, manual identification of disease spots and severity assessment is commonly used by planters; however, this process is labor-intensive and prone to human error (Liu et al., 2017).

In recent years, deep learning methods, particularly Convolutional Neural Networks (CNNs), have garnered significant attention in the field of plant disease recognition (Pal and Kumar, 2023; Beikmohammadi et al., 2022; Naik et al., 2022). Current research predominantly relies on single-background images (e.g., desktop, human palm) for recognition (He et al., 2024; Fatima Naqvi et al., 2024; Fu et al., 2024), as their stable backgrounds help highlight disease features, thereby improving recognition accuracy. However, single-background images face challenges in practical field applications, where it is often difficult to obtain backgrounds identical to those in the training images, which can lead to degraded model performance (Fu et al., 2024). Therefore, precise segmentation of diseased leaves to isolate them from complex and diverse backgrounds is crucial for enhancing the robustness and accuracy of recognition systems.

Image segmentation techniques, especially those leveraging advancements in deep learning, provide effective means to extract pepper leaves from images and are foundational for detecting and diagnosing diseases (Deb et al., 2022; Fang et al., 2021a; Ngugi et al., 2021). Traditional segmentation methods such as threshold-based and region-based techniques (Liu, 2012; Fang et al., 2021b) have been widely used but are limited by their reliance on image features and their inability to handle complex backgrounds effectively. Deep learning-based methods, especially CNNs and U-Net architectures, have shown promising results in semantic segmentation tasks (Long et al., 2015; Ronneberger et al., 2015; Li et al., 2018), but they often struggle with capturing detailed boundary information or handling multi-scale features.

Transformer-based networks (Dosovitskiy et al., 2020) have been proposed to address these issues by leveraging the self-attention mechanism, which allows for the extraction of global context information (Chen et al., 2021; Fang et al., 2023). However, many of these models focus primarily on global features and overlook detailed boundary information (Zhang et al., 2022). Multi-layer perceptron (MLP)-based networks, such as the MLP-Mixer (Tolstikhin et al., 2021), have recently demonstrated the potential to replace attention mechanisms, achieving competitive performance in image segmentation tasks by processing spatial information efficiently (Lv et al., 2022).

Building on these advancements, We present a novel approach for pepper leaf segmentation, called the adaptive multi-scale MLP (AMS-MLP) network. This network follows an encoder-decoder architecture, integrating the multi-path aggregation mask (MPAM) module with the multi-scale context relation decoder (MCRD) module. To enhance the fusion of global and local information between the encoder and decoder, we introduce the adaptive multi-scale MLP (AM-MLP) module, which replaces traditional skip connection layers. The AM-MLP module overcomes the limitations of convolutional layers’ inductive biases by effectively handling global information and progressively merging local details. Additionally, the MCRD module strengthens the model’s focus on foreground-background boundaries, especially around the segmented edges. Our contributions are as follows:

1. We propose a novel segmentation framework designed for accurate pepper leaf extraction from complex backgrounds. This framework outperforms previous methods by using a five-layer aggregation feature to generate a single-channel mask, improving segmentation precision along the pepper leaf boundaries.
2. We introduce the AM-MLP module, based on a self-attention mechanism, to automatically extract multi-scale features. This module consists of two branches: a Global Multi-scale MLP (GMS-MLP) branch and a Local Multi-scale MLP (LMS-MLP) branch, which capture global and local feature maps, respectively. The attention mechanism dynamically adjusts the weight assigned to each, ensuring effective fusion of both.
3. The MCRD module, leveraging an attention mechanism, combines features across adjacent scales, enhancing boundary delineation and contextual information for the segmented target.
4. Extensive experiments on the pepper leaf dataset demonstrate that our model outperforms state-of-the-art (SOTA) methods.

The remainder of the paper is structured as follows: Section 2 reviews related work on semantic segmentation methods. Section 3 details our network architecture. Section 4 describes the experimental setup, and Section 5 presents results and discussion. Finally, Section 6 concludes the paper.

## 2 Related works

### 2.1 Traditional semantic segmentation methods

Several traditional methods have been proposed for segmenting plant leaf images. Threshold-based techniques, such as fuzzy C-means algorithms (Liu, 2012), are commonly used to iteratively determine the optimal threshold for leaf image segmentation. Histogram-based thresholding methods, including bimodal histograms and Otsu’s Thresholding Method (Kalaivani et al., 2020; Fang et al., 2021b), have also been employed for segmenting leaf images. However, these threshold-based methods often struggle with complex images. Region-based approaches, such as the region-based level set method (Fang et al., 2021a), region growing methods (Jothiaruna et al., 2021), and wavelet methods (Xiong et al., 2020), have shown high accuracy and fast processing speeds for plant leaf segmentation. While these methods yield satisfactory results to some extent, their effectiveness is heavily dependent on image features, which limits their broader applicability. Clustering-based methods, such as fuzzy k-means clustering (Tian et al., 2019), have been used to determine cluster centers for leaf segmentation. However, these methods often struggle with local optima, leading to lower segmentation accuracy.

### 2.2 CNN-based models for semantic segmentation

Deep learning techniques have revolutionized the field of image segmentation, with convolutional neural networks (CNNs) playing a pivotal role. The introduction of fully convolutional networks (FCN) by Long et al. (2015) marked a significant milestone, replacing traditional fully connected layers with specialized convolutional layers tailored for segmentation tasks. Building on this, Ronneberger et al. (2015) proposed the U-Net architecture, which employs an encoder-decoder structure with skip connections to fuse low-level and high-level features. U-Net and its variants, such as R2U-Net (Alom et al., 2018) and BIONet (Xiang et al., 2020), have shown strong performance in segmentation, particularly for medical and agricultural applications. However, despite their success, CNN-based methods often face challenges in extracting detailed boundary information, especially in complex and varied environments.

To address these limitations, researchers have incorporated attention mechanisms into CNNs (Oktay et al., 2018; Zhou et al., 2019). For example, the squeeze-and-excitation network (SE-Net) (Hu et al., 2018) uses channel-wise attention to enhance global feature representation, while the attention-guided network (Li et al., 2019a) focuses on suppressing irrelevant background information. A parallel reverse attention network (PraNet) (Fan et al., 2020) introduced a reverse attention block to build relationships among object regions and boundaries. Despite their improvements, these models still struggle with precise boundary delineation, especially in complex segmentation tasks such as plant disease recognition.

### 2.3 Transformer-based models for semantic segmentation

Transformer-based models, originally designed for natural language processing (Devlin et al., 2018), have been adapted for computer vision tasks, including image segmentation. These models use self-attention mechanisms to capture long-range dependencies in images, improving segmentation accuracy for global features. For instance, TransUNet (Chen et al., 2021) combines the U-Net architecture with transformers to leverage high-level informative features for improved performance. Fang et al. (2023) proposed BAF-Net, a network combining CNNs and Swin Transformers for plant leaf segmentation. It utilizes MSFF and FSFF branches, enhanced by an adaptive bidirectional attention module, to capture comprehensive features. Dai et al. (2024) introduce AISOA-SSformer, a Transformer-based segmentation method for rice leaf disease detection. By integrating sparse global updates, feature attention, and optimized algorithms, it achieves high accuracy, aiding modern farming. However, transformer models often focus primarily on global context and struggle with capturing fine-grained details, such as object boundaries.

### 2.4 MLP-based models for semantic segmentation

Recently, multi-layer perceptron (MLP)-based models have gained attention as a viable alternative to CNNs and transformers for image segmentation. The MLP-Mixer (Tolstikhin et al., 2021) demonstrated that MLPs could replace self-attention mechanisms in image processing, achieving competitive performance in tasks like image classification. This idea was further explored in the Visual Transformer (ViT) (Melas-Kyriazi, 2021), where MLPs replaced the attention layers, showing that MLP-based networks could achieve similar results to CNNs and transformers in recognition tasks.

In segmentation tasks, MLP-based models like RepMLPNet (Ding et al., 2022) and MAXIM (Tu et al., 2022) have been shown to effectively replace self-attention mechanisms while maintaining high accuracy. These models utilize fully connected layers to capture both local and global context information, making them suitable for complex image segmentation tasks. Additionally, MLPs have been integrated with CNN architectures to form hybrid models that combine the benefits of both approaches. For instance, Valanarasu and Patel (2022) introduced UNeXt, a convolutional MLP-based network with a U-shaped architecture, comprising three convolution blocks and two tokenized MLP blocks for global information capture and pixel-wise classification. Similarly, the CM-MLP framework (Lv et al., 2022) integrates multi-scale feature interaction (MSFI) and axial context encoder (ACE) blocks, enhancing local information integration and establishing edge relations between foreground and background regions.

Inspired by the strengths of MLP-based models and transformers, our approach, the Adaptive Multi-Scale MLP (AMS-MLP), combines the benefits of both architectures to address these challenges. The AMS-MLP model integrates multi-path aggregation and multi-scale context relation modules, enabling dynamic fusion of global and local features for accurate segmentation, especially in complex backgrounds. To further highlight the novelty of our work, we provide a comprehensive comparison with existing leaf segmentation methods in **Table 1**. The table focuses on summarizing the Key Features, Strengths, and Limitations of existing methods, while explicitly outlining How AMS-MLP Differs/Improves over these approaches. Unlike previous approaches, AMS-MLP uniquely leverages adaptive multi-scale feature fusion and context-aware modeling, which significantly improves segmentation accuracy in challenging scenarios. This comparative analysis underscores the advancements of our method and its distinct contributions to the field of leaf segmentation.

**Table 1:** Comparison of our proposed AMS-MLP with existing leaf segmentation methods.

Category	Method	Key Features	Strengths	Limitations	How AMS-MLP Differs/Improves
Traditional Methods	Fuzzy C-means (Liu, 2012)	Iterative thresholding for segmentation	Simple and effective for basic images	Struggles with complex images; sensitive to noise	AMS-MLP uses dynamic feature fusion, handling complex backgrounds and noise robustly.
	Otsu’s Thresholding (Kalaivani et al., 2020)	Histogram-based thresholding	Works well for bimodal intensity distributions	Fails for images with overlapping intensity distributions	AMS-MLP leverages multi-scale context, overcoming intensity distribution challenges.
	Region Growing (Jothiaruna et al., 2021)	Region-based segmentation	High accuracy for simple leaf structures	Limited by seed point selection and image features	AMS-MLP does not rely on seed points; it adapts to varying leaf structures dynamically.
CNN-based Models	U-Net (Ronneberger et al., 2015)	Encoder-decoder with skip connections	Strong performance for medical and agricultural images	Struggles with detailed boundary information	AMS-MLP integrates multi-path aggregation for precise boundary delineation.
	SE-Net (Hu et al., 2018)	Channel-wise attention for global feature enhancement	Enhances global feature representation	Limited ability to suppress irrelevant background information	AMS-MLP uses adaptive bidirectional attention to focus on relevant regions and suppress noise.
	PraNet (Fan et al., 2020)	Reverse attention for object-boundary relationships	Improves object-boundary relationships	Struggles with fine-grained details in complex backgrounds	AMS-MLP combines multi-scale context and local-global feature fusion for fine-grained details.
Transformer-based Models	TransUNet (Chen et al., 2021)	Combines U-Net with transformers for global context	Captures long-range dependencies	Struggles with fine-grained details and boundary delineation	AMS-MLP integrates MLP-based local feature extraction with global context for better boundaries.
	BAF-Net (Fang et al., 2023)	Combines CNNs and Swin Transformers with adaptive bidirectional attention	Captures comprehensive features	Computationally expensive; struggles with fine details	AMS-MLP is computationally efficient and focuses on fine-grained details through multi-scale MLPs.
	AISOA-SSformer (Dai et al., 2024)	Transformer with sparse global updates and feature attention	High accuracy for rice leaf disease detection	Limited to specific applications; struggles with generalizability	AMS-MLP is generalizable and adaptable to various leaf segmentation tasks.
MLP-based Models	MLP-Mixer (Tolstikhin et al., 2021)	Replaces self-attention with MLPs for image processing	Competitive performance in image classification	Limited exploration in segmentation tasks	AMS-MLP specifically targets segmentation with multi-scale MLPs and dynamic feature fusion.
	UNeXt (Valanarasu and Patel, 2022)	Hybrid CNN-MLP with U-shaped architecture	Combines CNN and MLP benefits for segmentation	Limited ability to handle complex backgrounds	AMS-MLP enhances local-global feature integration and handles complex backgrounds effectively.
	CM-MLP (Lv et al., 2022)	Multi-scale feature interaction and axial context encoder	Improves local information integration	Struggles with edge relations in noisy images	AMS-MLP uses adaptive multi-scale context relations for robust edge detection.
Our Proposed Method	AMS-MLP	Combines MLP-based local feature extraction with global context fusion	Dynamic fusion of global and local features; robust to complex backgrounds	Requires careful tuning of multi-scale parameters	Novelty: Combines MLP and transformer strengths; adaptive multi-scale fusion for segmentation.

## 3 Methodology

### 3.1 Dataset

The pepper leaf image datasets utilized in this study were sourced from a private repository maintained by the Nanchang Academy of Agricultural Sciences. These datasets were collected from their farm located in Nanchang city, Jiangxi Province, China, specifically between August 12 and 13, 2022, using multi-view photography techniques. The camera used for image acquisition was equipped with an F5.6 lens and an EF-S 18-135mm f/3.5-5.6 IS USM microlens manufactured by Canon Company (Japan). During the data collection process, the camera was positioned at a height of 10-50 cm above the leaves to ensure high-resolution images. All images were captured after careful focusing, and the camera remained stationary during shooting to eliminate any motion blur or distortion caused by movement.

To ensure the comprehensiveness and practicality of the data, stringent inclusion criteria were established, encompassing various instances of pepper leaf diseases, including healthy leaves and those affected by viral infections. Specifically, the dataset includes images of leaves severely impacted by common diseases such as early blight, brown spot disease, and leaf mold, along with healthy pepper leaves (HPL) and viral diseases (VD) to enrich the diversity of the dataset. This hybrid database serves as a valuable resource for researching and developing methodologies related to pepper leaf segmentation and disease classification in agricultural research.

**Figure 1:**
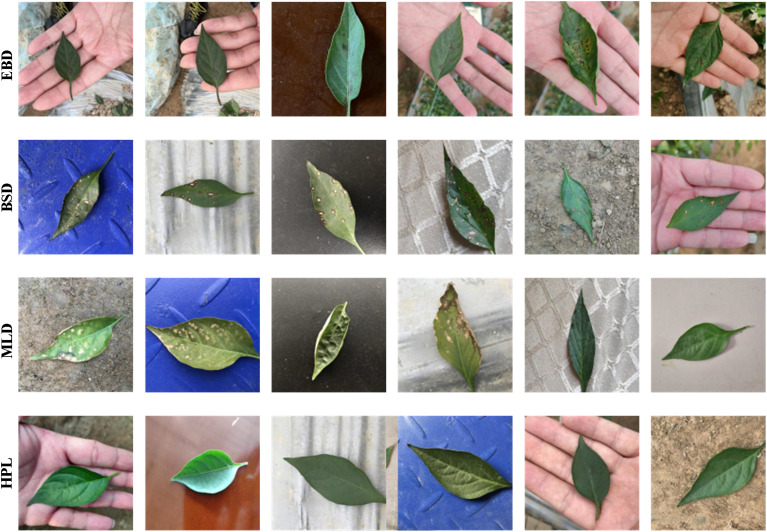
The sample images in different pure backgrounds.

To further evaluate the effectiveness of the proposed model in segmenting actual pepper leaves, four distinct datasets were meticulously constructed: Early Blight Dataset (EBD), Brown Spot Dataset (BSD), Leaf Mold Dataset (LMD), and Mixed Leaf Dataset (MLD). These datasets were manually annotated using the open-source tool LabelMe, assigning intensity values of 1 to foreground regions and 0 to background regions. During the data processing phase, we conducted a meticulous statistical analysis to ensure the representativeness and balance of the data across various disease categories and leaf conditions. This analysis involved calculating the distribution of images among different disease categories, conducting rigorous checks on the completeness and quality of annotations, and verifying the accuracy and reliability of the data. As shown in **Table 2**, the datasets for EBD, BSD,LMD, and MLD comprised 1190, 1384, 1385, and 6613 images, respectively. Notably, the MLD dataset integrates image data from EBD, BSD, LMD, as well as healthy pepper leaves and viral diseases, with 1353 images of healthy leaves and 1301 images of viral leaves. **Figure 1** shows several representative examples from the EBD, BSD, MLD and HPL, respectively. The statistical analysis confirmed the balanced nature of the MLD dataset, and all data were comprehensively annotated. Furthermore, to facilitate a comprehensive evaluation, each dataset was divided into training (70%), validation (10%), and testing (20%) subsets. In the experiment, we standardized the image size for each dataset to 512×512 pixels, facilitating consistent processing and analysis across all datasets.

**Table 2:** The distribution of the four image datasets.

Dataset	Test	Training	Validation	Total
**Early Blight Dataset (EBD)**	238	833	119	1190
**Brown Spot Dataset (BSD)**	277	970	138	1385
**Leaf Mold Dataset (LMD)**	277	969	138	1384
**Mixed Leaf Dataset(MLD)**	1323	4629	661	6613

Bold values indicate the best performance metrics in each category.

### 3.2 Method

In this section, we will provide an overview of the AMS-MLP model and discuss the incorporation of three key modules within the encoder-decoder architecture. These modules consist of the adaptive multi-scale MLP module, the multi-scale context relation decoder module, and the multi-path aggregation mask module. Additionally, we will present the loss function utilized in the model. By integrating these modules and utilizing an appropriate loss function, the AMS-MLP model demonstrates improved performance in image segmentation tasks.

#### 3.2.1 Overall architecture

**Figure 2:**
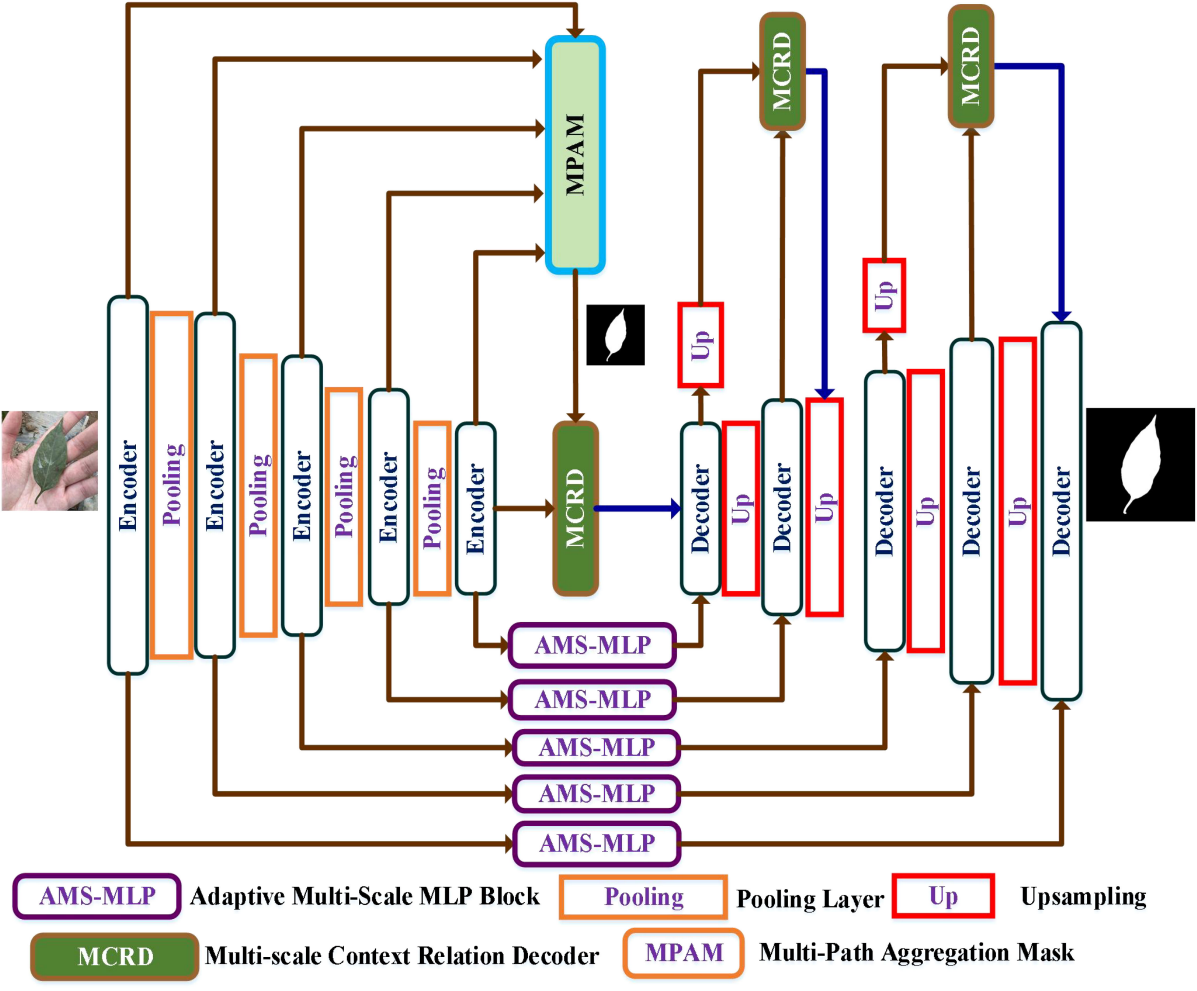
Overview of the AMS-MLP framework including the encoder network, the adaptive multi-scale MLP (AM-MLP) module, and the decoder network. The encoder network comprises five convolutional layers incorporating four downsampling operations and a multi-path aggregation mask (MPAM) module. The decoder network comprises five convolutional layers, incorporating four upsampling layers and three MSRD modules. The AM-MLP module is used for the skip connection layer.

**Figure 2** illustrates the network architecture of the proposed AMS-MLP network, based on a U-shape design. The AMS-MLP model consists of three core components: the encoder network, the AM-MLP module, and the decoder network. The encoder network includes five convolutional layers with four downsampling operations and integrates an MPAM module. Each convolutional block within the encoder comprises a 3×3 convolutional layer, batch normalization, ReLU activation, and max-pooling with a stride of 2. Multi-scale features from these layers are combined in the MPAM module to generate a preliminary mask, further refined by the MSRD module for edge information capture. The AM-MLP module, a critical component of the AMS-MLP network, employs self-attention to extract multi-scale features and local information automatically. The decoder network in the AMS-MLP model consists of five convolutional blocks with four upsampling layers and three MSRD modules. Each decoder block includes a 3×3 convolutional layer, batch normalization, and ReLU activation. The first MSRD module utilizes the mask from the MPAM module and features from the fifth layer, while subsequent MSRD modules further refine segmentation within the decoder. Deconvolution operations increase image resolution by a factor of 2 per block, restoring finer details lost during downsampling.

#### 3.2.2 Adaptive multi-scale MLP module

**Figure 3:**
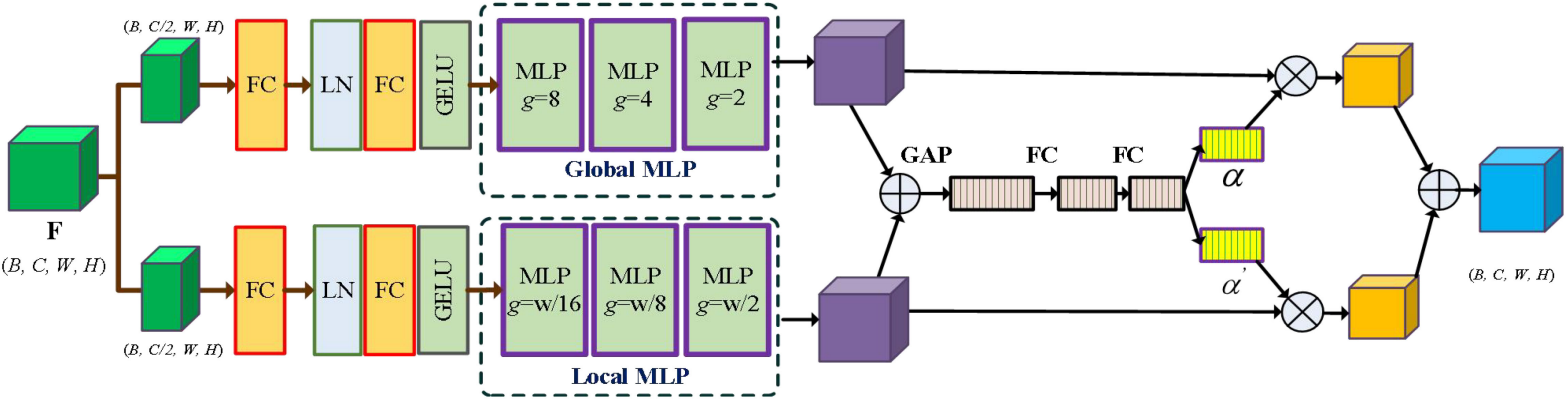
The network architecture of the AM-MLP module. The input feature map F is split into the global multi-scale MLP (GMS-MLP) branch **F^G^** and the local multi-scale MLP (LMS-MLP) branch **F^L^**. After each branch with multiple Cascade MLP blocks, the resulting features are alternately multiplied to enhance information interaction and then added together. Then, multi-scale features and local information are automatically extracted using an adaptive attention mechanism.

**Figure 4:**
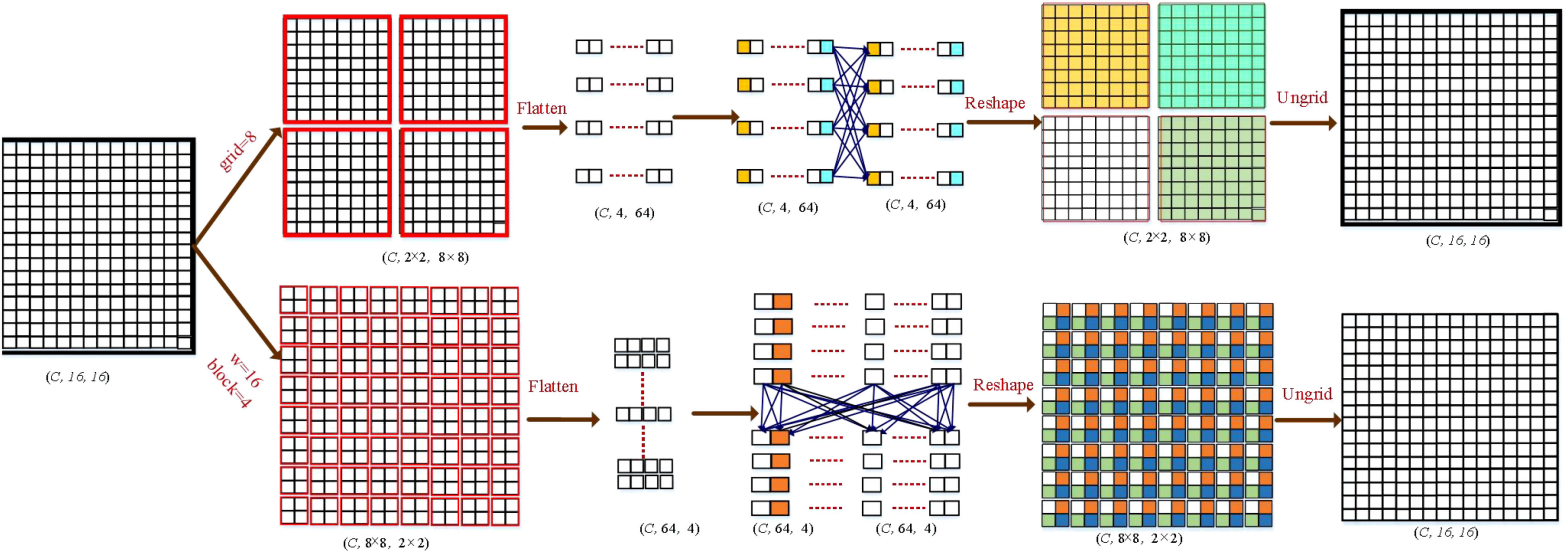
Illustration of the GMS-MLP and LMS-MLP modules. As an example, we used $F\in {ℜ}^{\left[\text{B},\text{ C},\text{ H},\text{ W}\right]}$ (W = 16; H = 16) as input, where B is the batch size, and C is the channel number. Input feature F will be processed by GMS-MLP and LMS-MLP branches. In the GMS-MLP branch, the feature map $F^{G}$ is initially divided into non-overlapping patches of size 2 × 2, resulting in a grid of size 8× 8. These patches are then flattened and fed into a fully connected (FC) layer along the first axis. Finally, the output is reshaped back and ungridded to restore the original size. In the LMS-MLP branch, the feature map $F^{L}$ is divided into non-overlapping patches of size 8 × 8, resulting in a blocking of size 2 × 2. These patches are flattened and processed through an FC layer along the second axis. Following that, the output is reshaped back and unblocked to regain the original size, resulting in the feature map $F_{mlp}^{L}$ .

The MLP module has demonstrated promising performance in the computer vision task, but it struggles with capturing spatial information and extracting global context due to its fully connected nature. To overcome these limitations, MAXIM (Tu et al., 2022) employs multi-scale MLP modules to extract global and local information. Inspired by MAXIM, we introduce an adaptive multi-scale MLP module that utilizes the self-attention mechanism to automatically extract multi-scale features and local information. As illustrated in **Figure 3**, the network initially splits the feature maps into two branches: the global multi-scale MLP (GMS-MLP) branch and the local multi-scale MLP (LMS-MLP) branch. The GMS-MLP branch focuses on extracting global features, while the LMS-MLP branch is dedicated to capturing local feature maps. **Figure 4** illustrates the GMS-MLP and LMS-MLP modules. To effectively combine these features, we introduce an adaptive attention module that dynamically adjusts the weights of the global and local features based on their importance and relevance to the task. By incorporating the adaptive multi-scale MLP module, the AM-MLP module enabled the extraction of both global and local information in an adaptive manner while preserving spatial information and capturing contextual cues from different scales.

Specially, the input features $F\in {ℜ}^{\left[\text{B},\text{ C},\text{ H},\text{ W}\right]}$ undergo an initial split into two branches based on the channel dimension, namely the GMS-MLP branch $F^{G}\in {\mathbb{R}}^{\left[\text{B},\text{ C}/2,\text{ H},\text{ W}\right]}$ and the LMS-MLP branch $F^{L}\in {ℜ}^{\left[\text{B},\text{ C}/2,\text{ H},\text{ W}\right]}$ , where B represents the batch size, C represents the channel number, and H and W represent the height and width of the image, respectively. In the GMS-MLP branch, the input features are first passed through a fully connected (FC) layer, followed by a layer normalization (LN) layer. The next step involves applying an additional fully connected (FC) layer and a GELU activation layer to generate the feature map $F_{fc}^{G}\in {ℜ}^{\left[\text{B},\text{ C}/2,\text{ H},\text{ W}\right]}$ . The generated feature map is then transformed into non-overlapping image patches, where each patch consists of a certain number of g×g grids. These patched features $F_{patch}^{G}\in {ℜ}^{\text{B},\text{ C},\text{ g}\times \text{g},\;{\text{H}}_{g}\times {\text{W}}_{g}}$ are further processed through three consecutive multi-scale MLP modules, where $\;{\text{H}}_{g}=\text{H}/\text{g},\;{\text{W}}_{g}=\text{W}/\text{g}$ , and g is the kernel size. This process leads to the generation of novel feature maps $F_{mlp}^{G}$ , which can be denoted as Equations 1–4:


(1) $$F^{G},\;F^{L}=split(F)\;F\in {ℜ}^{\text{B},\text{ C},\text{ H},\text{ W}},\;F^{G},\;F^{L}\in {ℜ}^{\text{B},\text{ C}/2,\text{ H},\text{ W}}$$

(2) $$F_{fc}^{G}=fc(Ln(fc(Gelu(F^{G}))))$$

(3) $$F_{patch}^{G}=Reshape(F_{fc}^{G})\;{\text{F}}_{\text{patch}}^{\text{G}}\in {ℜ}^{\text{B},\text{ C},\text{ g}\times \text{g},\;{\text{H}}_{g}\times {\text{W}}_{g}}$$

(4) $$F_{mlp}^{G}=mlp_{g}(F_{patch}^{G})\text{ g}\in [{\text{g}}_{1},\;{\text{g}}_{2},\;{\text{g}}_{3}]$$

where split(·) denoting dividing a multidimensional matrix or tensor into multiple sub-tensors along a channel dimension, and fc(·) denotes the full connection layer. Ln(·) denotes layer normalization layer, Gelu(·) denotes the GELU activation function, R eshape(·) denotes the operation of changing the shape or dimensions of two feature matrices. The GMS-MLP branch $mlp_{g}(\cdot )\;$ is three continuous MLP modules with the grid sizes of ${\text{g}}_{1}\times {\text{g}}_{1}$ , ${\text{g}}_{2}\times {\text{g}}_{2}$ , and ${\text{g}}_{3}\times {\text{g}}_{3}$ , respectively.

Similarly, in the LMS-MLP branch, the LMS-MLP feature $F^{L}$ passes through a FC layer, a layer normalization (LN) layer. Subsequently, it passes a FC layer and a GELU activation layer. The novel feature maps $F_{Fc}^{L}\in {ℜ}^{\left[\text{B},\text{ C}/2,\text{ H},\text{ W}\right]}$ are projected into non-overlapping image patches and generate a new feature maps $F_{block}^{L}\in {ℜ}^{\text{B},\text{ C},\text{ b}\times \text{b},\;{\text{H}}_{b}\times {\text{W}}_{b}}$ , where $\;{\text{H}}_{b}=\text{H}/\text{b},\;{\text{W}}_{b}=\text{W}/\text{b}$ , b is the kernel size, and the size of each image patch is b×b grids. Then, the feature maps $F_{fc}^{L}$ pass three continuous multi-scale MLP modules to obtain the spatial information, which is written as Equations 5–7:


(5) $$F_{fc}^{L}=fc(Ln(fc(Gelu(F^{L}))))$$

(6) $$F_{block}^{L}=Reshape(F_{fc}^{L})\;{\text{F}}_{\text{block}}^{\text{L}}\in {ℜ}^{\left[\text{B},\text{ C},\text{ b}\times \text{b},\;{\text{H}}_{b}\times {\text{W}}_{b}\right]}$$

(7) $$F_{mlp}^{L}=mlp_{\text{b}}(F_{block}^{L})\text{ b}\in [{\text{b}}_{1},{\text{b}}_{2},{\text{b}}_{3}]$$

where $mlp_{b}(\cdot )$ is three continuous MLP modules with the grid sizes of ${\text{b}}_{1}\times {\text{b}}_{1}$ , ${\text{b}}_{2}\times {\text{b}}_{2}$ , and ${\text{b}}_{3}\times {\text{b}}_{3}$ , respectively.

A self-attention module is employed to effectively fuse two features $F_{mlp}^{L}$ and $F_{mlp}^{L}$ obtained from the GMS-MLP and LMS-MLP branches, and it guides the segmented network to select more representative features from the channel dimension. Specially, two features $F_{mlp}^{\text{G}}$ and $F_{mlp}^{\text{L}}$ are fused, and followed by the global average pooling (GAP) operation to compress the channel dimension, which can be represented as follows Equation 8:


(8) $$F_{\text{H}}^{G}=GAP(F_{mlp}^{\text{G}}⊕F_{mlp}^{\text{L}})$$

where $F_{H}^{G}$ is the output features of the GAP layer. Then, the features $F_{H}^{G}$ are input into a FC layer, followed by a batch normalization layer, and a softmax function. The probability feature maps $F_{H}^{FC}$ can be expressed as Equation 9:


(9) $$F_{\text{H}}^{\text{FC}}=\sigma (BN(fc(F_{\text{H}}^{G})))$$

where σ(·) denotes the sigmoid activation function, and BN(·) is a batch normalization layer. Then, we perform another FC layer on the features $F_{H}^{FC}$ followed by the softmax activation function, and the channel attention map α is written as Equation 10:


(10) $$\alpha =\sigma_{sf}(fc(F_{\text{H}}^{\text{FC}}))$$

where $\sigma_{sf}(\cdot )$ denotes the softmax activation layer. We regard the channel attention map α∈[0, 1] as the weight of the features, where $\alpha \in {ℜ}^{C\times 1\times 1}$ . The channel attention map $\alpha^{'}\in [0,\;1]$ is from the value α , and it satisfies $\alpha^{'}=1-\alpha$ . An important observation is that the channel attention maps α and $\alpha^{'}$ enable the adaptive adjustment of weights for the two channel attention feature maps. It also demonstrates that the two feature maps are capable of extracting feature representations from different receptive fields. By flexibly adjusting the adaptive weights of two features $F_{Fc}^{G}$ and $F_{Fc}^{L}$ , the feature maps can be expressed as Equations 11–13:


(11) $$F_{G}^{'}=\alpha \times F_{FC}^{G}$$

(12) $$F_{L}^{'}=\alpha^{'}\times F_{FC}^{L}$$

(13) $$F^{out}=F_{G}^{'}⊙F_{L}^{'}$$

where ⊙ denotes the concatenation operator, $F_{\text{G}}^{'},\;F_{\text{L}}^{'}\in {ℜ}^{\left[\text{B},\text{ C}/2,\text{ H},\text{ W}\right]}$ are two output features from the adaptive dot-product features, respectively.

Notably, the grid size **g** and the block size **b** satisfy a specific relationship. As exemplified in **Figure 3**, the network structure of the GMS-MLP and LMS-MLP branches is depicted. When reducing the patch size in the GMS-MLP block, the block size in the LMS-MLP branch increases accordingly. For instance, when considering an image size of 32, the grid sizes in the GMS-MLP branch are set to 8, 4, and 2, while the corresponding grid sizes in the LMS-MLP branch are 4, 8, and 16, respectively. This arrangement results in a larger number of patches within the global MLP, enabling the capture of spatial information among the patches. Conversely, in the LMS-MLP branch, a larger number of pixels in each block allows for the retention of local spatial information between pixels. Consequently, by fusing the GMS-MLP and LMS-MLP blocks, a comprehensive feature map can be generated, encompassing both global and local information in a progressively richer manner.

#### 3.2.3 Multi-scale context relation decoder module

**Figure 5:**
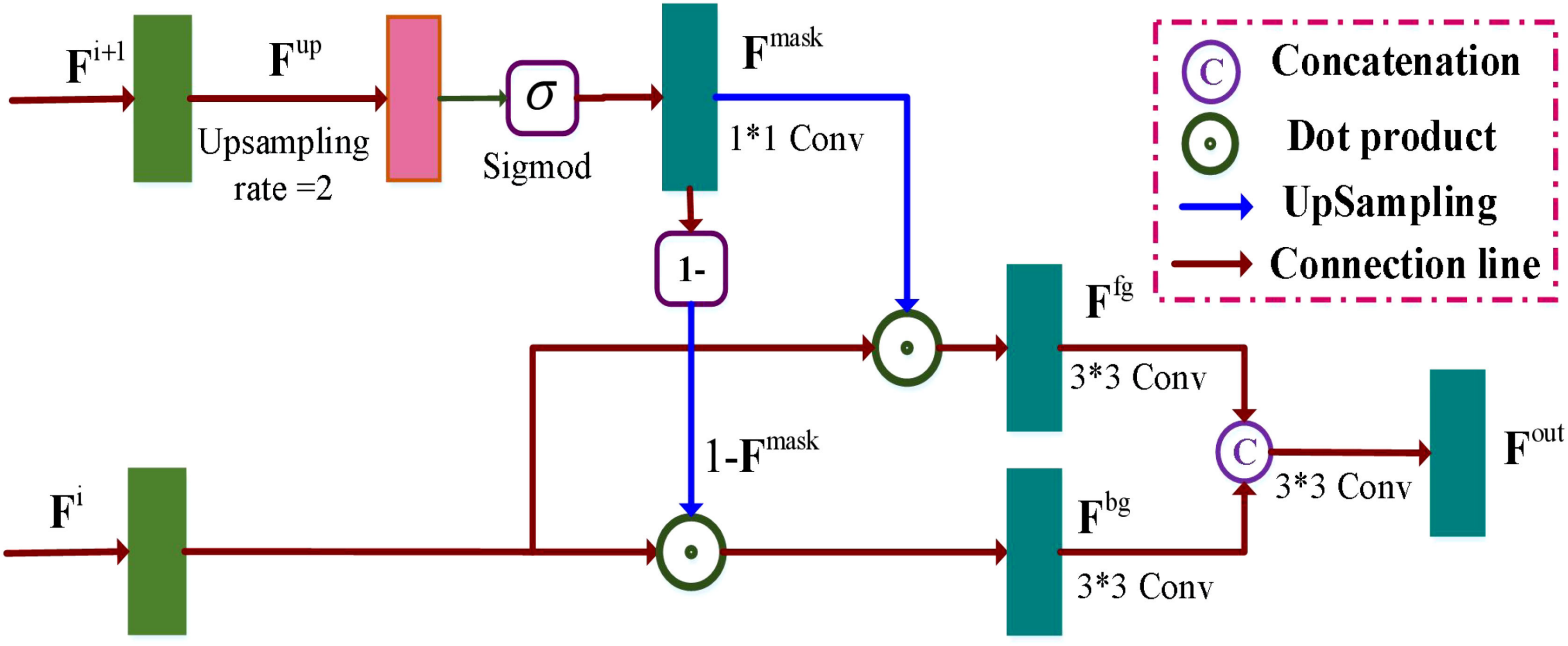
Illustration of the multi-scale context relation decoder (MCRD) module. Two feature maps $F^{\text{i}}$ and $F^{\text{i}+1}$ are input into the MCRD module, the high features is first performed on the upsampling operation. The generated feature maps $F^{\text{up}}$ pass through the sigmoid activation function and a 1×1 convolutional operation, which generates the mask maps $F^{\text{mask}}$ representing the foreground and background regions.


The accurate extraction of boundaries between foreground and background regions relies on the presence of both local and contextual information. To address this, the Mask refinement network (Tang et al., 2021) leverages contextual relationships to improve the pixel boundaries in these regions. In line with this, we propose an MCRD module to enhance the target boundary features and contextual information. As shown in **Figure 5**, our approach involves initial upsampling of the high feature maps $F^{\text{i}+1}$ through non-linear interpolation with a rate of 2, followed by a sigmoid activation function. The novel feature maps are then fed into a 1×1 convolutional block, which generates an output with a single channel. The process is formulated as Equations 14, 15:

(14) $$F^{\text{up}}=up(F^{\text{i}+1})$$

(15) $$F^{\text{mask}}=Conv_{1\times 1}(\sigma (F^{\text{up}}))$$

where up(·) denotes the upsampling operator, $Conv_{1\times 1}(\cdot )$ is a 1×1 convolutional operation.

Then, the mask maps $F^{\text{mask}}$ is used to assign different weights of the foreground and background feature maps, which are written as Equations 16, 17:


(16) $$F^{\text{fg}}=Conv_{3\times 3}(F^{\text{i}}⊗F^{\text{mask}})$$

(17) $$F^{\text{bg}}=Conv_{3\times 3}(F^{\text{i}}⊗(1-F^{\text{mask}}))$$

where ⊗ denotes the dot product, $Conv_{3\times 3}(\cdot )$ is a 3×3 convolutional block.

Finally, we concatenate two feature maps $F^{\text{fg}}$ and $F^{\text{bg}}$ on the channel dimension, and it then perform a 3×3 convolutional layer, which is written as Equation 18


(18) $$F^{\text{bg}}=Conv_{3\times 3}(F^{\text{fg}}⊙F^{\text{bg}})$$

#### 3.2.4 Multi-path aggregation mask module

**Figure 6:**
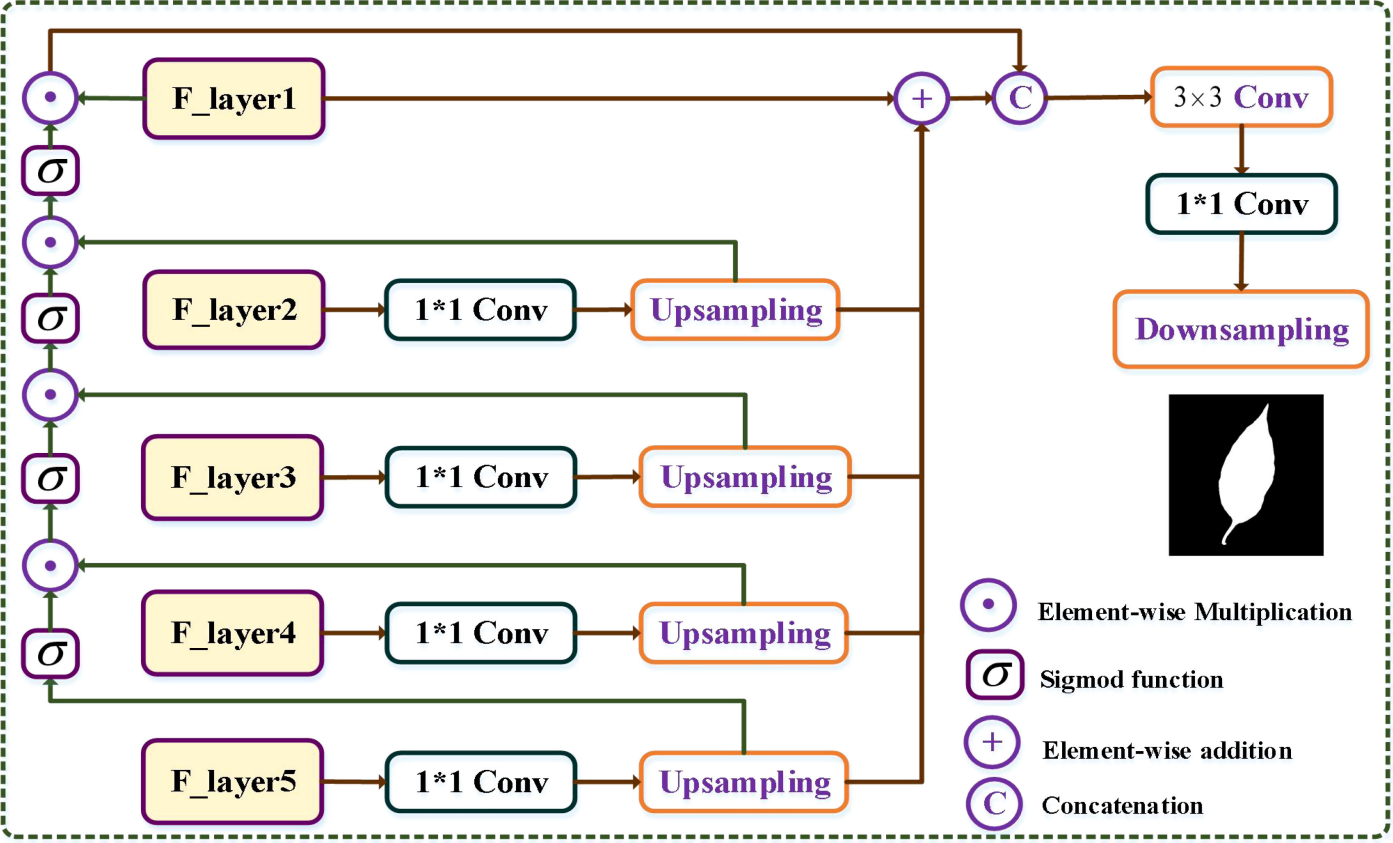
Illustration of the multi-path mask decoder module. From the fifth to second layers, the feature maps $F_{\text{i}}$ in the encoder are first passing a 1×1 convolutional operation to suppress the channel number, and the generated channel number of the output features is the same to that of the first layer in the encoder.


The multi-scale nature of features in deep neural networks offers different levels of information, with deeper layers capturing coarser details and shallower layers preserving finer details. To leverage the benefits of each layer, we introduce an MPAM module to enhance the extraction of accurate boundary information and facilitate the generation of masks. As shown in **Figure 6**, for the feature map $F_{\text{i}}$ from the fifth layer to the second layer in the encoder, each feature map is subjected to a 1×1 convolutional operation to decrease the channel dimensions. The resulting feature maps have the same channel number as the first layer in the encoder. Additionally, we employ an upsampling operation with a rate of 2 on these feature maps. This procedure can be expressed as Equation 19:

(19) $$F_{\text{i}-1}^{\text{up}}=up(Conv_{1\times 1}(F_{\text{i}}))\text{ i}=2,\cdots 5$$

Subsequently, the generated feature maps are further processed by the Sigmoid activation function. We then concatenate the generated feature $F_{\text{i}-1}^{\text{up}}$ and the previous feature maps $F_{\text{i}-1}$ , and the final feature maps are written as Equation 20:


(20) $$F_{\text{i}-1}^{\text{out}}=\sigma (F_{\text{i}-1}^{\text{up}})⊙\;F_{\text{i}-1}\text{ i}=2,\cdots 5$$

To incorporate information from various scales, we utilize an element-wise addition operation between four upsampling feature maps and the feature maps $F_{\text{i}-1}^{\text{up}}$ obtained from the first layer in the encoder. This operation produces multi-scale fusion feature maps (MSFF), which can be denoted as Equation 21:


(21) $$F^{\text{cat}}={\left\{F_{\text{i}}^{\text{up}}⊙\right\}}_{\text{i}=1}^{4}\;F_{1}$$

In the final step, we concatenate the MSFF maps $F^{\text{cat}}\;$ with the feature maps $F_{1}^{\text{out}}$ obtained from the first layer. The concatenated feature maps $F_{1}^{\text{en}}$ are then fed into a convolutional block with 3×3 filters. To generate the mask for the foreground and background regions, we apply a 1×1 convolutional operation with a single output channel, followed by a downsampling operation. Mathematically, this can be represented as Equations 22, 23:


(22) $$F_{1}^{\text{en}}=F^{\text{cat}}⊙\;F_{1}^{\text{out}}$$

(23) $$F_{\text{i}-1}^{\text{up}}=dn(Conv_{3\times 3}(F_{\text{i}}))\text{ i}=2,\cdots 5$$

where dn(·) denotes the downsampling operator.

## 4 Experimental setup

### 4.1 Experimental environment configuration

The proposed AMS-MLP network was implemented on a specific hardware configuration consisting of a 12th Gen Intel(R) Core(TM) i7-12700K 3.60 GHz processor and an NVIDIA GeForce RTX 3090 40 GB GPU with 32 GB of RAM. The operating system employed was Windows 11, and the Conda environment was utilized to ensure a consistent software environment for the execution.

### 4.2 Experimental scheme

For the parameter settings of the AMS-MLP network, we employed a batch size of 2 and trained the model for a total of 60 epochs. The optimization process was performed using stochastic gradient descent (SGD) with an initial learning rate of 0.001, and the learning rate was adjusted according to the learning rate schedule. During training, these parameter choices facilitated effective convergence of the AMS-MLP network. To assess the model’s performance, we compared it against several state-of-the-art (SOTA) models using various performance metrics. Additionally, ablation studies were conducted to evaluate the impact of different network components, such as the choice of activation functions and the depth of layers, on the overall performance. In our experiments, we also tested other values for batch size, learning rate, and epoch count, but found that the chosen configuration yielded the best performance in terms of both training stability and testing accuracy.

### 4.3 Training loss

The proposed AMS-MLP network involves two loss functions to optimize the predicted result and the ground truth (GT), including the binary cross entropy (BCE) $L_{b}$ and the Dice $L_{d}$ . The two loss functions are defined as Equations 24, 25:


(24) $$L_{b}(f,g)=-\sum_{i=1}^{N}\left[g_{x}\log(f_{x})+(1-g_{x})\log(1-f_{x})\right]$$

(25) $$L_{d}(f,g)=1-\frac{2\sum_{i=1}^{N}f_{x}\cdot g_{x}}{\sum_{i=1}^{N}f_{x}+\sum_{i=1}^{N}g_{x}}$$

where *f* denotes the input predicted result, and *g* denotes the corresponding ground truth label.

Therefore, our final loss $L_{loss}$ can be expressed as Equation 26:


(26) $$L_{loss}(f,g)=\alpha L_{bce}(f,g)+L_{d}(f,g)$$

### 4.4 Performance evaluation

To rigorously evaluate the performance of the proposed method and other compared methods, six metrics are employed as evaluation criteria: accuracy, recall, precision, specificity, F1-score, and intersection over union (IoU). Here’s a detailed breakdown of these metrics. These metrics are defined as follows:

#### 4.4.1 Accuracy

Accuracy measures the overall correctness of the predictions, calculated as the ratio of correctly predicted pixels (both foreground and background) to the total number of pixels.

#### 4.4.2 Recall

Recall (also known as sensitivity) measures the ability of the model to identify all relevant instances (foreground pixels), calculated as the ratio of true positives (TP) to the sum of true positives and false negatives (FN).

#### 4.4.3 Precision

Precision measures the accuracy of the positive predictions, calculated as the ratio of true positives to the sum of true positives and false positives (FP).

#### 4.4.4 Specificity

Specificity measures the ability of the model to identify all irrelevant instances (background pixels), calculated as the ratio of true negatives (TN) to the sum of true negatives and false positives.

#### 4.4.5 F1-score

F1-score is a harmonic mean of precision and recall, providing a single metric that balances both the precision and the recall of the model. It is particularly useful when the classes are of unequal size or when there is a trade-off between precision and recall.

#### 4.4.6 Intersection over Union

Intersection over Union (IoU) is a metric commonly used to evaluate the accuracy of boundary predictions. It measures the overlap between the predicted border and the real border by calculating the ratio of their intersection to their union.

These metrics are standard measures widely used for performance evaluation, and they are defined as Equations 27–31:


(27) $$\text{accuracy}=\frac{TP+TN}{TP+TN+FP+FN}$$

(28) $$\text{recall}=\frac{TP}{TP+FN}$$

(29) $$\text{precision}=\frac{TP}{TP+FP}$$

(30) $$\text{specificity}=\frac{TN}{TN+FP}$$

(30) $$F1=\frac{2\times PR\cdot PP}{PR+PP}$$

(31) $$IoU=\frac{TP}{TP+FP+FN}$$

where TP (true positive) represents the number of pixels that are correctly predicted as foreground, TN (true negative) indicates the number of pixels that are correctly predicted as background, FP (false positive) refers to the number of pixels that are predicted as foreground but actually belong to the background according to the ground truth. On the other hand, FN (false negative) represents the number of pixels that are predicted as background but actually belong to the foreground according to the ground truth.

## 5 Results and discussion

### 5.1 Comparison with the SOTA models

To evaluate the segmentation performance of the AMS-MLP model, we conducted a comparative study against state-of-the-art (SOTA) models using three distinct leaf datasets: EBD, BSD, and MLD. The models included FCN-VGG16, U-Net (Ronneberger et al., 2015), attention U-Net (AttU-Net) (Oktay et al., 2018), UNet++ (Zhou et al., 2019), UNeXt (Valanarasu and Patel, 2022), and CM-MLP model (Lv et al., 2022). To ensure a fair and comprehensive comparison, all models were trained, validated, and tested on the same three datasets. By maintaining consistency across the training, validation, and test datasets, we aim to eliminate any potential bias or variation that may affect the results and evaluate the segmentation performance exclusively on the test dataset.

**Figure 7:**
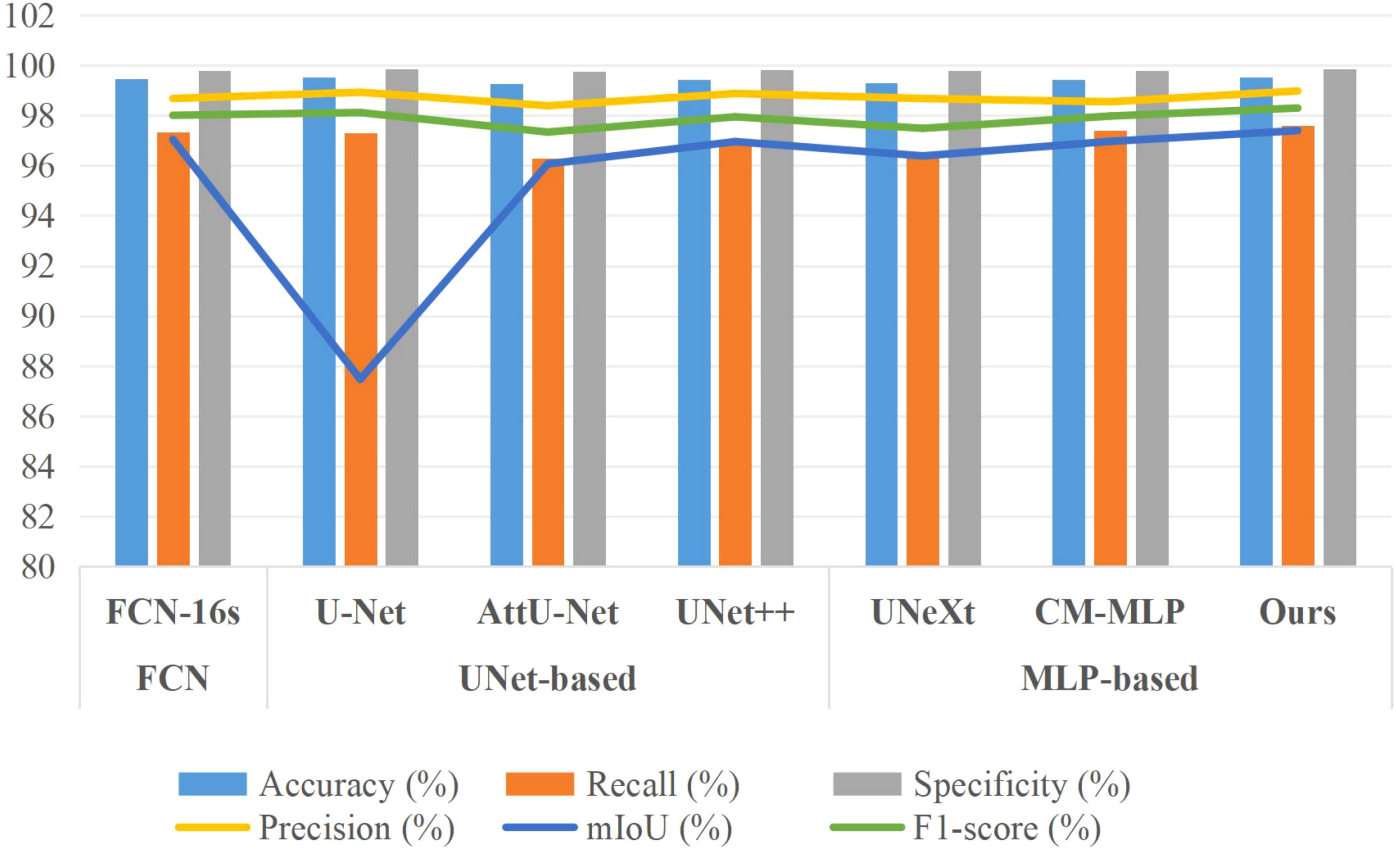
Bar chart comparison of seven models’ performance on the EBD dataset.

**Figure 8:**
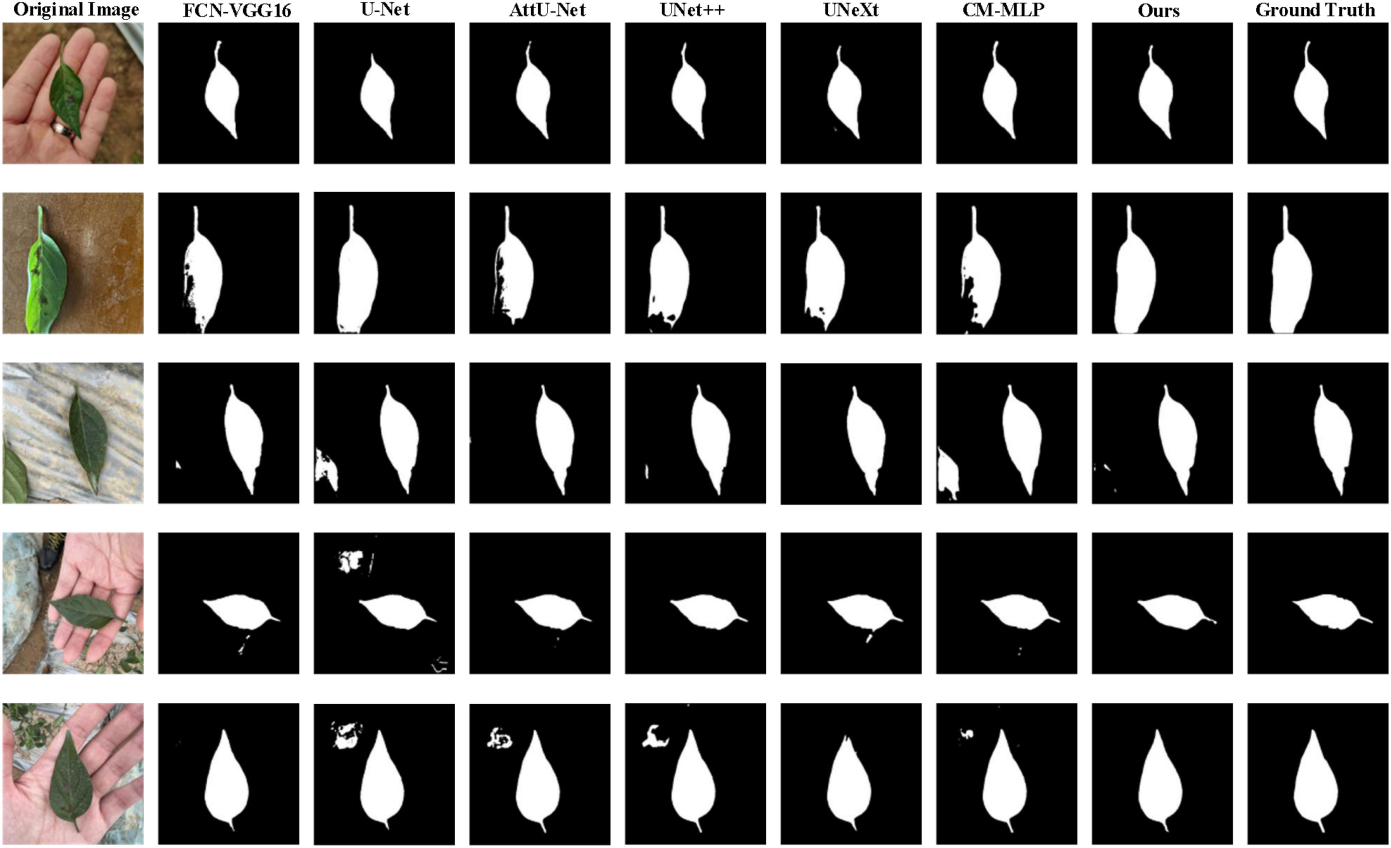
Qualitative comparison of the proposed model compared with six models on the EBD dataset, and five examples of the predicted results are shown. From the 1^st^ column to 9^th^ column: the original image, the predicted results corresponding to FCN-VGG16, U-Net, AttUNet, UNet++, UNeXt, CM-MLP, our model, and the ground truth, respectively.

**Table 3** and **Figure 7** present the segmentation results of the AMS-MLP model compared to seven other models on the EBD dataset, evaluated across six metrics: accuracy, recall, precision, mean Intersection over Union (mIoU), and F1-score. Our model achieves the highest scores across five of these metrics, notably achieving 97.39% in mIoU and 98.29% in F1-score, surpassing FCN by 0.35% and 0.29%, respectively. These superior metrics reflect the AMS-MLP model’s ability to leverage the GMS-MLP and LMS-MLP modules effectively. The GMS-MLP module captures global context, enabling robust feature extraction across the entire image, while the LMS-MLP module enhances local detail recognition within specific leaf structures. This dual-stream approach ensures comprehensive information integration, leading to enhanced segmentation accuracy. Compared to U-Net, our model demonstrates significant improvements with increases of 9.92% in mIoU, 0.18% in F1-score, and 0.29% in recall. This enhancement can be attributed to the AMS-MLP’s capacity to combine both global and local features effectively, thereby improving boundary delineation and reducing segmentation errors. Furthermore, compared to other semantic segmentation models, our approach achieves the highest scores in accuracy, recall, precision, mIoU, and F1-score, underscoring its robust performance across multiple evaluation criteria. Qualitative examples in **Figure 8** highlight the models’ ability to locate object regions accurately, with our proposed model notably delineating pepper leaf boundaries with precision. This visual evidence further substantiates the effectiveness and superiority of the AMS-MLP model in leaf segmentation tasks.

**Table 3:** The results of segmenting the EBD dataset using seven different models.

Model		Accuracy (%)	Recall (%)	Specificity (%)	Precision (%)	mIoU (%)	F1-score (%)
**FCN**	**FCN-16s**	99.45	97.33	99.79	98.67	97.04	98.00
**UNet-based**	**U-Net**	99.53	97.31	**99.85**	98.92	87.47	98.11
	**AttU-Net**	99.26	96.29	99.74	98.38	96.06	97.33
	**UNet++**	99.43	97.04	99.82	98.87	96.95	97.94
**MLP-based**	**UNeXt**	99.31	96.31	99.79	98.67	96.38	97.48
	**CM-MLP**	99.44	97.41	99.77	98.54	96.96	97.97
	**Ours**	**99.53**	**97.61**	99.84	**98.97**	**97.39**	**98.29**

Bold values indicate the best performance metrics in each category.

**Figure 9:**
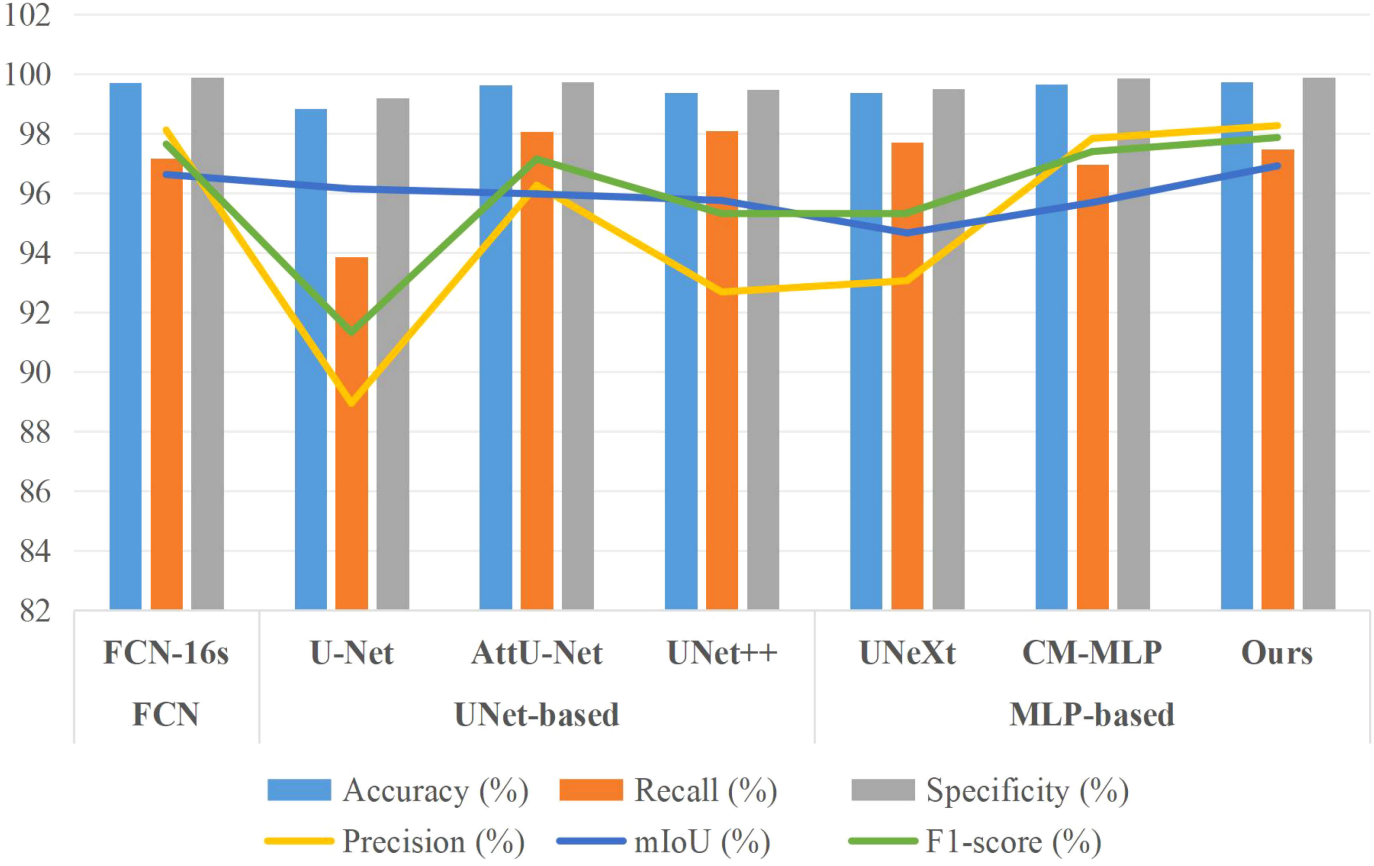
Bar chart comparison of seven models’ performance on the BSD dataset.

**Figure 10:**
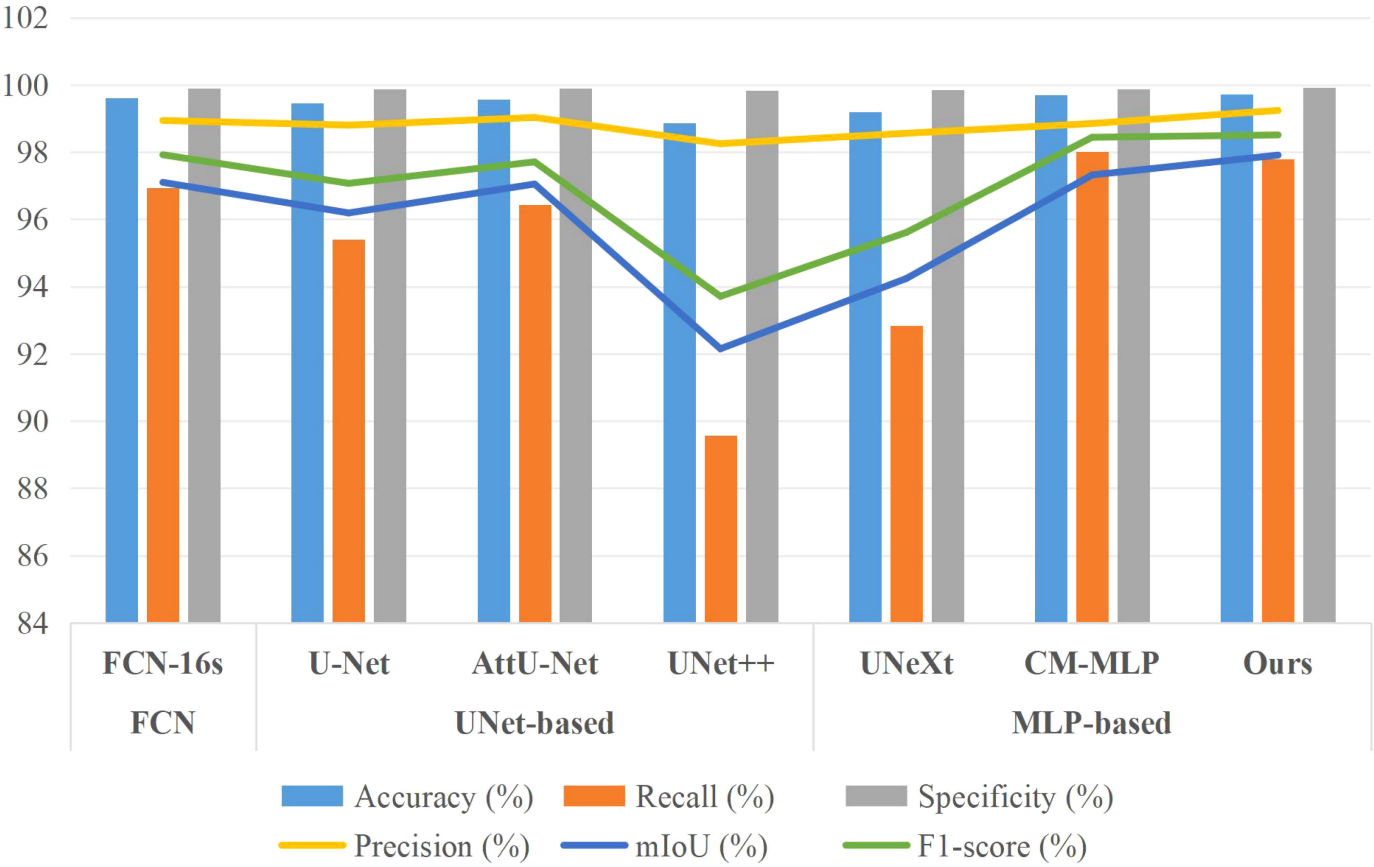
Bar chart comparison of seven models’ performance on the MLD dataset.

Additionally, for a comprehensive evaluation of training performance, we conducted a comparative analysis of the AMS-MLP network against FCN-based, U-Net-based, and additional MLP-based models, utilizing the BSD and MLD datasets as benchmarks. The results of this comparison are presented in **Tables 4**, **5**, and **Figures 9**, **10**. Our method consistently outperformed other models across five evaluation metrics. This improvement can be attributed to the integration of GMS-MLP and LMS-MLP modules, enabling the extraction of both global and local information crucial for enhancing segmentation accuracy. The FCN-16s model’s superior performance over U-Net is attributed to its use of pretrained VGG16, which enriches feature extraction and representation within the encoder. Similarly, the CM-MLP model, by leveraging MLP instead of traditional attention mechanisms, achieves superior segmentation results by effectively considering pixel relationships.

**Table 4:** The results of segmenting the BSD dataset using seven different models.

Model		Accuracy (%)	Recall (%)	Specificity (%)	Precision (%)	mIoU (%)	F1-score (%)
**FCN**	**FCN-16s**	99.69	97.17	99.87	98.11	96.62	97.64
**UNet-based**	**U-Net**	98.83	93.85	99.18	88.95	96.14	91.33
	**AttU-Net**	99.62	98.05	99.73	96.26	95.97	97.14
	**UNet++**	99.37	**98.08**	99.46	92.68	95.75	95.31
**MLP-based**	**UNeXt**	99.37	97.70	99.49	93.06	94.66	95.33
	**CM-MLP**	99.66	96.95	99.85	97.83	95.68	97.39
	**Ours**	**99.72**	97.47	**99.88**	**98.26**	**96.91**	**97.86**

Bold values indicate the best performance metrics in each category.

**Table 5:** The results of segmenting the MLD dataset using seven different models.

Model		Accuracy (%)	Recall (%)	Specificity (%)	Precision (%)	mIoU (%)	F1-score (%)
**FCN**	**FCN-16s**	99.61	96.93	99.89	98.94	97.10	97.92
**UNet-based**	**U-Net**	99.46	95.40	99.88	98.80	96.19	97.07
	**AttU-Net**	99.57	96.43	99.90	99.03	97.05	97.71
	**UNet++**	98.87	89.58	99.83	98.25	92.15	93.71
**MLP-based**	**UNeXt**	99.20	92.84	99.86	98.56	94.24	95.61
	**CM-MLP**	99.71	**98.02**	99.88	98.85	97.32	98.44
	**Ours**	**99.72**	97.79	**99.92**	**99.24**	**97.91**	**98.51**

Bold values indicate the best performance metrics in each category.

**Figure 11:**
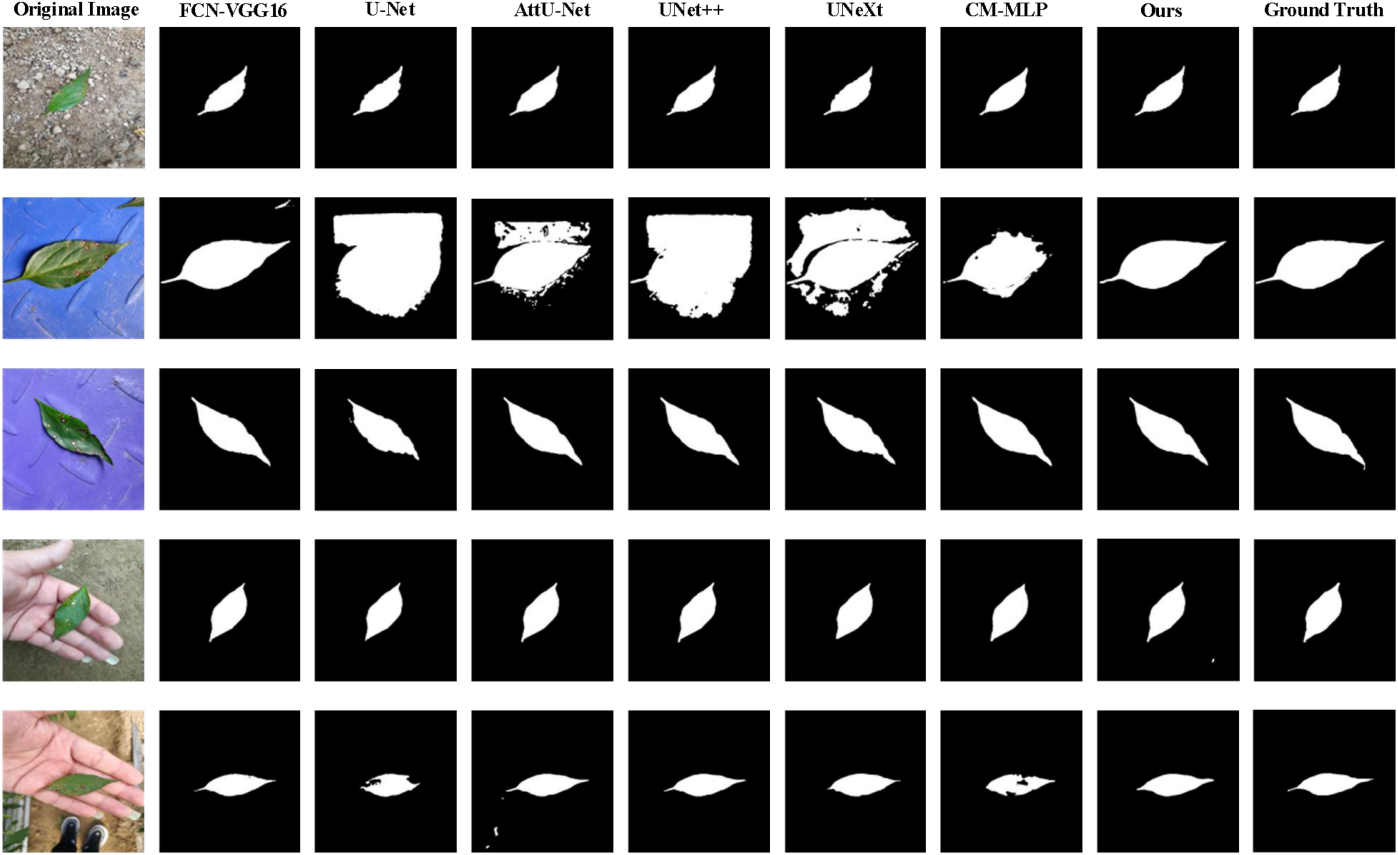
Qualitative comparison of the proposed model compared with six models on the BSD dataset, and five examples of the predicted results are shown. From the 1^st^ column to 9^th^ column: the original image, the predicted results corresponding to FCN-VGG16, U-Net, AttUNet, UNet++, UNeXt, CM-MLP, our model, and the ground truth, respectively.

**Figure 11**, **12** provide visual insights into segmentation outputs, revealing that U-Net and UNet++ models exhibit certain false positive regions in lesion segmentation. In contrast, AttU-Net shows improved performance over U-Net, while the AMS-MLP model closely approximates ground truth, demonstrating precise extraction of pepper leaf boundaries and reduced false positive regions. This performance superiority is facilitated by the MCRD module and the utilization of GMS-MLP and LMS-MLP auxiliary streams, which facilitate effective cascaded contraction and expansion processes within the network.

**Figure 12:**
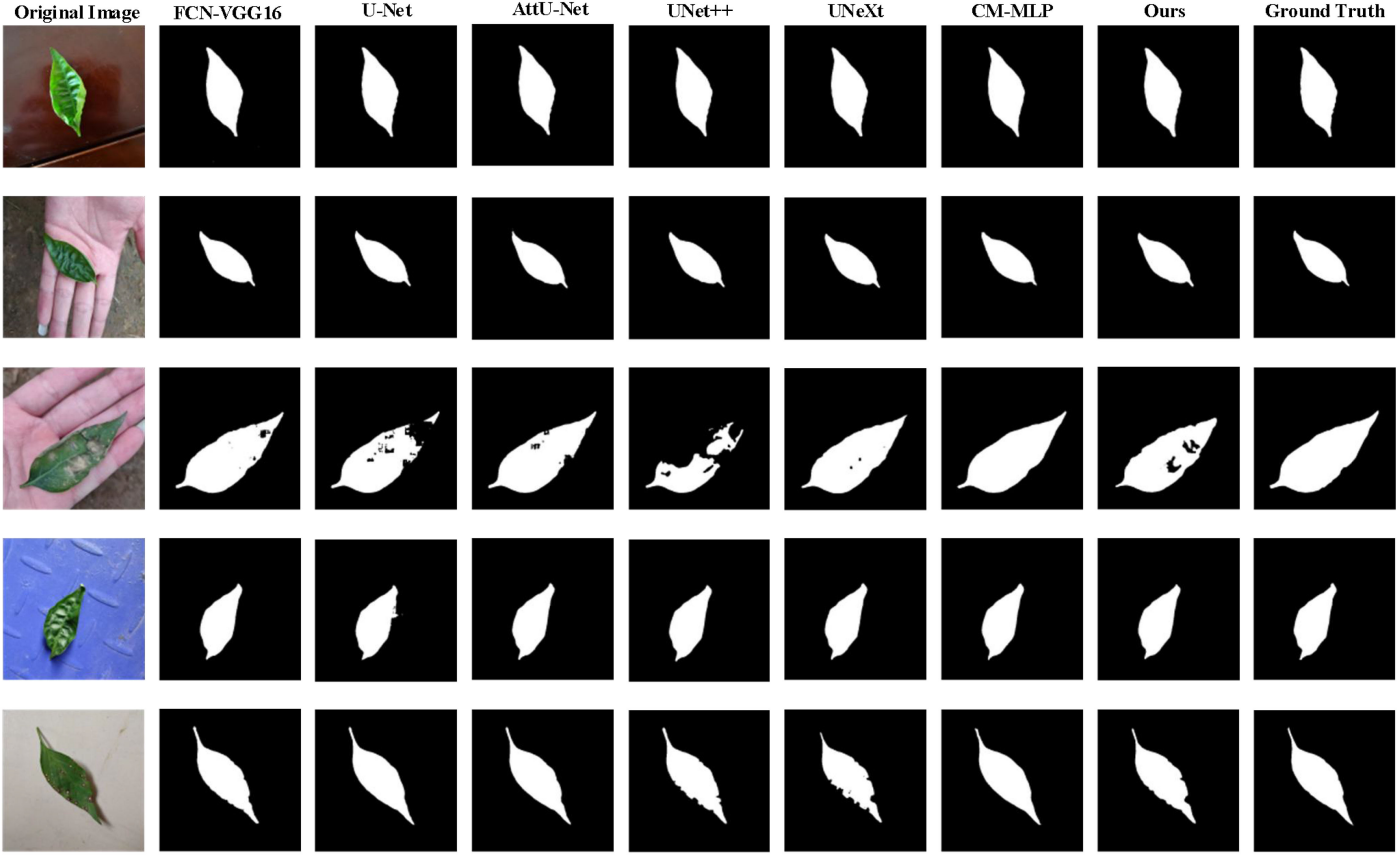
Qualitative comparison of the proposed model compared with six models on the MLD dataset, and five examples of the predicted results are shown. From the 1^st^ column to the 9^th^ column: the original image, the predicted results corresponding to FCN-VGG16, U-Net, attention U-Net (AttUNet), UNet++, UNeXt, CM-MLP, our model, and the ground truth, respectively.

Despite these advancements, our study acknowledges areas for further improvement, particularly in optimizing computational efficiency for real-time applications and scaling the model to larger datasets. Specifically, we recognize that, in terms of model size, our proposed AMS-MLP does not demonstrate a significant advantage compared to other models, such as CM-MLP, FCN-16s, U-Net, AttU-Net, and UNet++, as evidenced by our analysis of memory footprint and storage requirements. Nevertheless, we believe our method retains value in other critical aspects, such as potentially offering higher accuracy or efficiency in specific data processing scenarios. Future research will focus on refining architectural designs, exploring advanced training strategies, and actively working on optimizing our method to address these challenges and further elevate segmentation performance. Our ongoing efforts include strategies to reduce resource consumption and enhance overall performance, aiming to better meet practical application needs.

### 5.2 Ablation study

**Figure 13:**
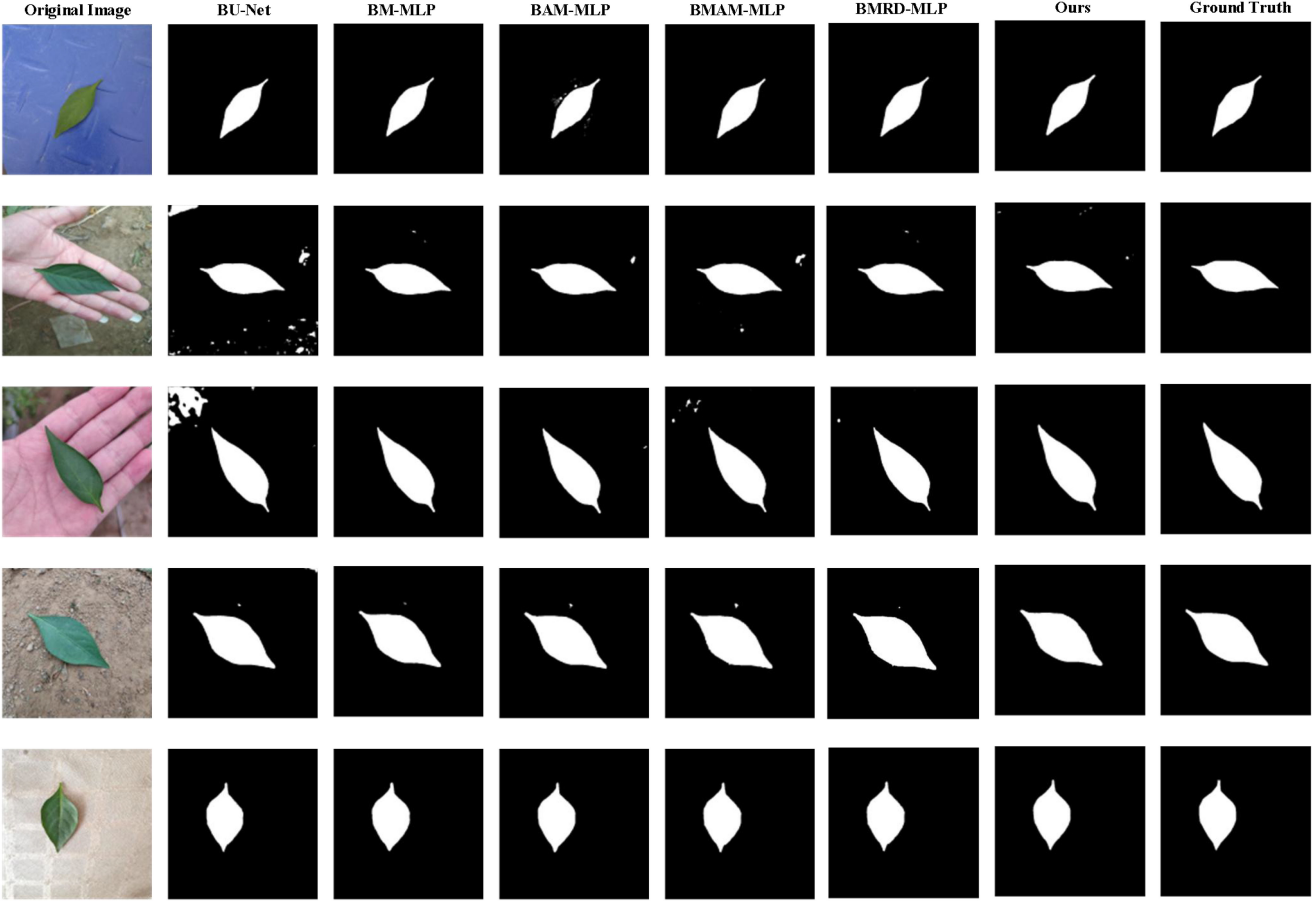
Qualitative comparison for the ablation study on the MLD dataset, and six predicted results are shown. From the 1^st^ column to 8^th^ column: the original image, the predicted results corresponding to BU-Net, BM-MLP, BAM-MLP, BMAM-MLP, BMRD-MLP, our model, and the ground truth, respectively.

In this section, we conducted a comprehensive ablation study to systematically evaluate the impact of individual modules on segmentation performance using the MLD dataset. Our baseline model was derived from the BU-Net architecture, with a modified channel configuration to reduce model complexity. To assess the contribution of each module, we adopted a phased integration approach. Initially, we introduced the BAM-MLP model by incorporating the AM-MLP module into the BU-Net architecture. The AM-MLP module enhances the network’s ability to capture global context information, which improves the focus on informative regions, thereby boosting segmentation performance. Subsequently, we integrated the MPAM module into the encoder, specifically at the fifth layer, resulting in the BMAM-MLP model. The MPAM module is crucial for generating precise masks, refining the segmentation process by better delineating object boundaries. Further, to explore the synergistic effects of incorporating multiple modules, we tested the BMRD-MLP model, which integrates both the AM-MLP and MCRD modules into the BU-Net architecture. The MCRD module enhances the network’s ability to preserve boundary details, improving segmentation accuracy by focusing on fine-grained features. Finally, we developed the AMS-MLP model by progressively combining the AM-MLP, MPAM, and MCRD modules. This multi-module integration demonstrated superior performance across multiple evaluation metrics, illustrating the complementary effects of these modules in enhancing segmentation accuracy. Through this incremental approach, we were able to systematically assess the contribution of each module. As shown in **Figure 13**, the results clearly demonstrate the positive impact of each module on enhancing the overall segmentation performance.

**Table 6** presents the detailed results of all ablation experiments. As illustrated in **Table 5**, our study commenced with the BU-Net model, which achieved the following performance metrics: 97.65% accuracy, 93.92% recall, 79.11% precision, 97.96% specificity, 97.74% mIoU, and 85.88% F1-score. To improve upon this baseline, we incorporated the AM-MLP module into the BU-Net architecture, resulting in the BAM-MLP model. A comparative analysis revealed that BAM-MLP outperformed BU-Net across all metrics, achieving 98.37% accuracy, 96.70% recall, 84.20% precision, 98.51% mIoU, and 90.02% F1-score—improvements of 0.72%, 2.78%, 0.55%, 0.32%, and 4.14%, respectively. Building on these results, we investigated the synergistic effects of adding more modules to the BAM-MLP model. We first integrated the MPAM module, leading to the BMAM-MLP model, and then included the MCRD module, resulting in the BMRD-MLP model. Both modifications brought about substantial improvements in segmentation performance, with noticeable gains in accuracy, recall, precision, specificity, and F1-score compared to the BAM-MLP model. Lastly, we constructed the AMS-MLP network by incorporating both the MPAM and MCRD modules into the BAM-MLP architecture. The MPAM and MCRD modules effectively harnessed multi-scale features, preserving boundary details and further enhancing segmentation performance. The integration of these three modules—AMSS-MLP, MPAM, and MCRD—yielded the best overall performance, as demonstrated by a thorough analysis of the combined metrics. This analysis clearly highlights the distinct contributions of each module when applied to the MLD dataset.

**Table 6:** The Compared results for the ablation experiment of pepper leaf segmentation.

Model	AM-MLP	MPAM	MCRD	Accuracy (%)	Recall (%)	Specificity (%)	Precision (%)	mIoU (%)	F1-score (%)
**Baseline (BU-Net)**				97.65	93.92	79.11	97.96	97.74	85.88
**BAM-MLP**	✓			98.37	96.70	84.20	98.51	98.06	90.02
**BMAM-MLP**	✓	✓		99.04	96.78	91.36	99.25	96.88	93.87
**BMRD-MLP**	✓		✓	98.90	97.02	92.10	99.28	98.18	94.29
**Ours**	✓	✓	✓	**99.63**	**97.98**	**97.20**	**99.77**	**98.28**	**97.59**

Bold values indicate the best performance metrics in each category. √ denotes the module is included in the model architecture for this ablation variant.

### 5.3 Real-world applications for ground-based mobile disease recognition

The primary goal of this study is to develop a precise pepper leaf segmentation model to support ground-based mobile disease recognition systems. The proposed Adaptive Multi-Scale MLP (AMS-MLP) model is specifically designed to segment diseased leaves from images captured under various pure backgrounds (e.g., palm, ground, or desktop), which are commonly used in ground-based data collection scenarios. By accurately extracting leaves from complex backgrounds, the segmented leaves can be fed into disease recognition models, significantly improving their accuracy and robustness.

The AMS-MLP model is optimized for deployment on mobile devices (e.g., smartphones), making it highly accessible and practical for small-scale farmers. In real-world applications, farmers or agricultural workers can use a smartphone to capture images of pepper leaves placed on different pure backgrounds in the field. The AMS-MLP model will then precisely segment the leaves, enabling subsequent disease recognition models to perform more effectively. This approach is particularly beneficial for farmers who rely on mobile devices for crop monitoring and disease detection, as it provides a cost-effective and user-friendly solution.

While our current focus is on ground-based mobile applications, the AMS-MLP model can also be adapted for other platforms, such as UAVs (drones) or robotic systems, in future work. For example, integrating the model into drones could enable large-scale monitoring of pepper fields, while embedding it into agricultural robots could support automated disease detection and precision farming. However, the immediate application of our research is to enhance the performance of mobile-based disease recognition systems by improving leaf segmentation accuracy under varying backgrounds.

## 6 Conclusion

Accurate extraction of plant leaves from diverse backgrounds is of significant importance for building robust plant disease recognition models. In this study, we propose a lightweight and high-precision leaf segmentation model specifically designed for extracting pepper leaves in complex and variable backgrounds. The model adopts an encoder-decoder architecture, innovatively integrating an Adaptive Multi-scale MLP (AM-MLP) network, a Multi-scale Pyramid Aggregation Module (MPAM), and a Multi-channel Residual Decoding (MCRD) module. In the encoder, the MPAM module enhances the accuracy of leaf edge feature extraction through cross-layer feature aggregation and single-channel masking. The AM-MLP module employs a dual-branch structure: the Global Multi-scale MLP (GMS-MLP) branch extracts global contextual features, while the Local Multi-scale MLP (LMS-MLP) branch generates local feature maps and optimizes feature representation through a dynamic attention mechanism. The decoder integrates the MCRD module, leveraging convolutional layers to improve boundary localization capabilities. The results demonstrate that the proposed method exhibits excellent robustness and generalization capabilities, achieving mean Intersection over Union (mIoU) scores of 97.39%, 96.91%, and 97.91%, as well as F1 scores of 98.29%, 97.86%, and 98.51%, respectively. Ablation studies further confirm that the progressive integration of the AM-MLP, MPAM, and MCRD modules significantly improves the model’s performance across six key evaluation metrics.

Despite the outstanding performance of the proposed AMS-MLP network in pepper leaf segmentation tasks, certain limitations remain. First, the model relies entirely on supervised learning, requiring a large amount of precisely annotated training data. Second, there is still room for optimization in computational efficiency. Based on these observations, we outline the following future research directions: (1) exploring weakly supervised and self-supervised learning methods to reduce dependency on annotated data; (2) investigating model fine-tuning strategies to enhance generalization across different scenarios; (3) further optimizing computational efficiency to meet real-time processing requirements on ground mobile devices; (4) extending the model to platforms such as drones and robotic systems; and (5) improving network architecture design and exploring advanced training strategies to further enhance segmentation performance. These research directions will provide more efficient and versatile solutions for plant leaf segmentation in complex backgrounds.

## Data Availability

The raw data supporting the conclusions of this article will be made available by the authors, without undue reservation.
